# Quantum‐Capacitance Biosensing Enables Real‐Time Monitoring of Naxitamab for Therapeutic Response Stratification in Neuroblastoma

**DOI:** 10.1002/smsc.70349

**Published:** 2026-07-31

**Authors:** Andy Bruno, Ruslán Alvarez‐Diduk, Paula Lara, Gabriel Maroli, Cristina Larrosa, Sandra López‐Miralles, Jaume Mora, Carlos J. Rodríguez‐Hernández, Arben Merkoçi

**Affiliations:** ^1^ Universitat Autonoma de Barcelona (UAB) Barcelona Spain; ^2^ Catalan Institute of Nanoscience and Nanotechnology (ICN2), CSIC and BIST Barcelona Spain; ^3^ Pediatric Cancer Center Barcelona (PCCB) Hospital Sant Joan de Déu Esplugues de Llobregat Spain; ^4^ Pediatric Cancer Institut de Recerca Sant Joan de Déu (IRSJD) Esplugues de Llobregat Spain; ^5^ Catalan Institution for Research and Advanced Studies (ICREA) Barcelona Spain

**Keywords:** label‐free quantum‐capacitance biosensor, laser‐assisted, naxitamab, neuroblastoma, rGO‐based composite, single‐point calibration, therapeutic drug monitoring

## Abstract

Neuroblastoma, a leading cause of cancer‐related mortality in early childhood, relies on anti‐GD2 immunotherapy, whose efficacy is critically dependent on systemic drug exposure that remains largely unmonitored in real time, limiting treatment optimization and early response assessment. Here, a first‐in‐class, label‐free electrochemical biosensing platform is reported for the direct quantification of naxitamab in patient‐derived samples. The system is based on a laser‐assisted reduced graphene oxide–gold nanoparticle (rGO@AuNPs) nanocomposite, enabling ultrasensitive detection through quantum capacitance modulation arising from perturbations in the electronic density of states of graphene upon biomolecular recognition. Laser‐assisted fabrication yields nanostructured electrodes with enhanced surface area and conductivity, promoting stable antibody immobilization and reproducible signal transduction. The platform operates in the femtomolar regime, with a linear response between 25 and 300 fM, high specificity against relevant interferents, and robust performance in diluted human serum. A single‐point calibration strategy enables accurate quantification while compensating for device variability. Application to retrospective clinical samples reveals distinct post‐infusion concentration profiles, enabling early discrimination between responder groups and detection of antidrug antibody development. This work establishes quantum‐capacitance biosensing as a generalizable strategy for real‐time monitoring of therapeutic antibodies in precision oncology.

## Graphical abstract

Quantum‐capacitance biosensing enables real‐time monitoring of naxitamab in patient samples via laser‐fabricated electrodes and single‐point calibration. The proposed platform provides rapid and sensitive quantification, with the potential to enable therapeutic drug monitoring and patient stratification according to circulating naxitamab levels, while supporting real‐time assessment of drug exposure through a simple, robust, and clinically translatable electrochemical sensing approach for future clinical applications.

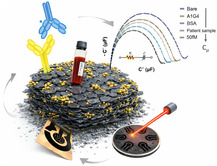

## 1 Introduction

Neuroblastoma is a malignant pediatric cancer originating from neural crest cells, which give rise to components of the sympathetic nervous system. Predominantly affecting children under the age of five, the disease is marked by considerable heterogeneity in both clinical presentation and biological behavior. Despite the integration of surgery, chemotherapy, and radiation therapy into multi‐modal treatment protocols, the prognosis for high‐risk cases remains poor, with long‐term survival rates falling below 50% [1]. In response, immunotherapy has emerged as a promising strategy to improve clinical outcomes, particularly through the development of monoclonal antibodies targeting disialoganglioside GD2, a glycolipid highly expressed on the surface of neuroblastoma cells. These antibodies offer a critical advantage over traditional therapies, enabling selective targeting of malignant cells while sparing normal tissues [2].

Among the most clinically advanced of these immunotherapies is naxitamab (Danyelza), a newly FDA‐approved humanized antibody that binds GD2. Through this interaction, naxitamab initiates immune‐mediated cytotoxicity via antibody‐dependent cellular cytotoxicity (ADCC) and complement‐dependent cytotoxicity (CDC), promoting tumor cell elimination [1]. Clinical trials have demonstrated its efficacy in patients with refractory or relapsed neuroblastoma, particularly when administered in combination with chemotherapeutics or other immunomodulatory agents. However, the clinical efficacy of naxitamab critically depends on achieving and maintaining adequate circulating antibody levels [2, 3, 4, 5, 6].

Despite these advances, several challenges hinder the optimal use of naxitamab in clinical settings. Current quantification of circulating naxitamab relies on centralized enzyme‐linked immunosorbent assays (ELISA), which are inherently incompatible with real‐time therapeutic decision‐making [6, 7]. This limitation prevents dynamic dose adjustment, delays the identification of nonresponding patients, and hinders early detection of treatment‐neutralizing immune responses. Furthermore, many pediatric patients experience severe infusion‐related pain, attributed to GD2 expression in peripheral nerve tissues, making dose adjustment an urgent clinical priority [8].

Compounding these issues, a subset of patients mounts a humoral immune response against naxitamab, leading to the production of human antihuman antibodies (HAHAs). These antibodies could neutralize the therapeutic agent, rendering the treatment ineffective and forcing discontinuation [8]. Monitoring naxitamab levels in real time could serve as an indirect method for early detection of HAHAs, allowing clinicians to adjust or suspend therapy before treatment failure occurs.

Given the central role of naxitamab in managing high‐risk neuroblastoma, the development of a rapid, sensitive, and clinically deployable detection platform is of critical importance. Here, a first‐in‐class electrochemical biosensing platform is reported for the direct and ultrasensitive quantification of naxitamab in clinically relevant samples, enabling real‐time assessment of therapeutic exposure and response. The system is implemented as a label‐free electrochemical biosensor based on reduced graphene oxide electrodes embedded with gold nanoparticles (AuNPs), functionalized with the monoclonal antibody A1G4, which exhibits high affinity for naxitamab [7]. The integration of two‐dimensional materials with metal NPs has been shown to substantially enhance sensing technologies, enabling the development of high‐performance biosensors and smart diagnostic platforms. A one‐step laser nanostructuring method has been employed to fabricate reduced graphene oxide films embedded with AuNPs, generating scalable and efficient sensing interfaces. This strategy allows the instantaneous laser co‐reduction of graphene oxide and gold precursor, resulting in conductive rGO nanosheets with uniformly distributed AuNPs. The resulting hybrid structures have demonstrated remarkable sensitivity and selectivity for the detection of analytes such as caffeic acid, nitrite, and hydrogen peroxide, in addition to biomarkers including CA‐19‐9 glycoprotein and pathogenic *Escherichia coli* [9, 10, 11, 12]. In this context, the fabrication strategy was tailored to ensure robustness and reproducibility under clinically relevant conditions, enabling reliable operation in patient‐derived samples.

The detection mechanism relies on quantum capacitance, a powerful transduction principle that enables real‐time, high‐resolution monitoring of target–receptor interactions without the need for additional labeling. This electrochemical strategy is based on a label‐free approach. The method leverages the unique electrical properties of the material to detect changes in quantum capacitance upon antibody–antigen binding, providing a highly sensitive and specific detection mechanism without the need for additional labeling steps [11]. This approach simplifies the detection process and enhances the biosensor's practicality for clinical applications. Quantum capacitance arises from the quantum mechanical properties of the electrode material, particularly those with low‐dimensional structures like graphene. Unlike classical capacitance, which is dominated by the electrostatic storage of charge, quantum capacitance is determined by the density of states (DOS) near the Fermi level of the material [13, 14, 15]. In materials such as reduced graphene oxide, the quantum capacitance (*C*_q_) is a function of the change in the number of electronic states available for occupation near the Fermi energy (*E*_f_):

$$C_{q}=e^{2}D(E_{f})$$

where *e* is the electron charge, and *D*(*E*_f_) is the DOS at the Fermi level [16].

In electrolyte‐gated systems, the experimentally measured capacitance corresponds to the electrochemical capacitance (*C*_µ_), which arises from the series combination of the ionic (electrostatic) capacitance (*C*_*i*_) and the quantum capacitance (*C*_q_), following the relationship $1/C_{\mu }=1/C_{i}+1/C_{q}$. In the operational framework commonly adopted in quantum‐capacitance biosensing, the ionic or electrostatic capacitance is assumed to be significantly larger than the quantum contribution, leading to the approximation *C*_µ_ ≈ *C*_q_ [11, 13, 14, 15]. More recently, an isoscopic description has been proposed for electrolyte–screened rGO interfaces, in which electrostatic and quantum capacitances are considered energetically degenerate (*C* *_i_ *= *C*_q_), yielding $C_{\mu }=C_{q}/2$ [17]. Within this framework, active participation of the electrolyte in the transduction process is assumed through electric‐field screening of electronic states. Although this refined description provides additional physical insight and may be relevant for the future optimization of electrolyte‐gated sensing platforms, the relative capacitance variations employed for quantification in the present work are not affected because the proportionality factor is preserved across all measurements performed under identical electrolyte conditions. Accordingly, the electrochemical capacitance measured in the present study remains directly linked to variations in the electronic DOS of the sensing interface.

The binding of naxitamab to the A1G4‐functionalized electrode alters the local electronic environment at the electrode surface. This interaction modifies the DOS of the composite material, leading to changes in its quantum capacitance. The detection mechanism, therefore, relies on monitoring variations in quantum capacitance arising from antigen–antibody interactions. As the concentration of naxitamab increases, a progressive reduction of the semicircle in the complex capacitance plane is observed, indicating a corresponding decrease in the quantum capacitance of the system. The quantum capacitance is obtained from the low‐frequency limit of the real component of the complex capacitance, determined by fitting the capacitance spectra as the frequency approaches zero.

Notably, the quantum‐capacitance transduction mechanism also enables the implementation of an internal single‐point calibration strategy. In contrast to previously reported quantum‐capacitive biosensors that rely on multipoint calibration curves [13, 15, 18, 19], the signal can be referenced to the intrinsic electronic baseline of the graphene electrode, enabling quantitative detection through a single calibration point. This simplified analytical approach could facilitate translation toward point‐of‐care diagnostics and, in the long term, enable more accessible monitoring technologies that bring therapeutic control closer to the patient.

The biosensor demonstrated excellent performance in detecting naxitamab in human serum samples, accurately distinguishing nonresponders, defined as patients with negligible circulating antibody in samples collected 5 min after infusion, from strong responders. By providing timely and precise data, this platform addresses critical gaps in current clinical workflows, establishing a framework for real‐time therapeutic monitoring of monoclonal antibodies in precision oncology. Importantly, this capability enables early response stratification based on post‐infusion exposure, supports the development of exposure‐driven therapeutic strategies, and provides a potential route for the indirect detection of immunogenicity through deviations in circulating antibody levels.

## 2 Results and Discussion

### 2.1 Electrode Preparation and Characterization

The development of the electrochemical platform was grounded on a fabrication methodology previously established within our research group, serving as the structural basis for the present study [9, 10, 11, 12, 20]. The electrode was fabricated using a laser‐assisted co‐reduction strategy for the generation of rGO@AuNPs nanocomposites [9, 11]. Briefly, graphene oxide (GO) films were prepared by vacuum filtration in the presence of an aqueous gold salt precursor. Subsequent laser etching using a CO_2_ laser facilitated the simultaneous reduction of GO to rGO through localized photothermal effects and promoted the in situ nucleation and growth of AuNPs, yielding patterned rGO@AuNPs electrodes with integrated conductive networks. These patterned films were then transferred onto PET substrates preprinted with silver conductive tracks.

**FIGURE 1:**
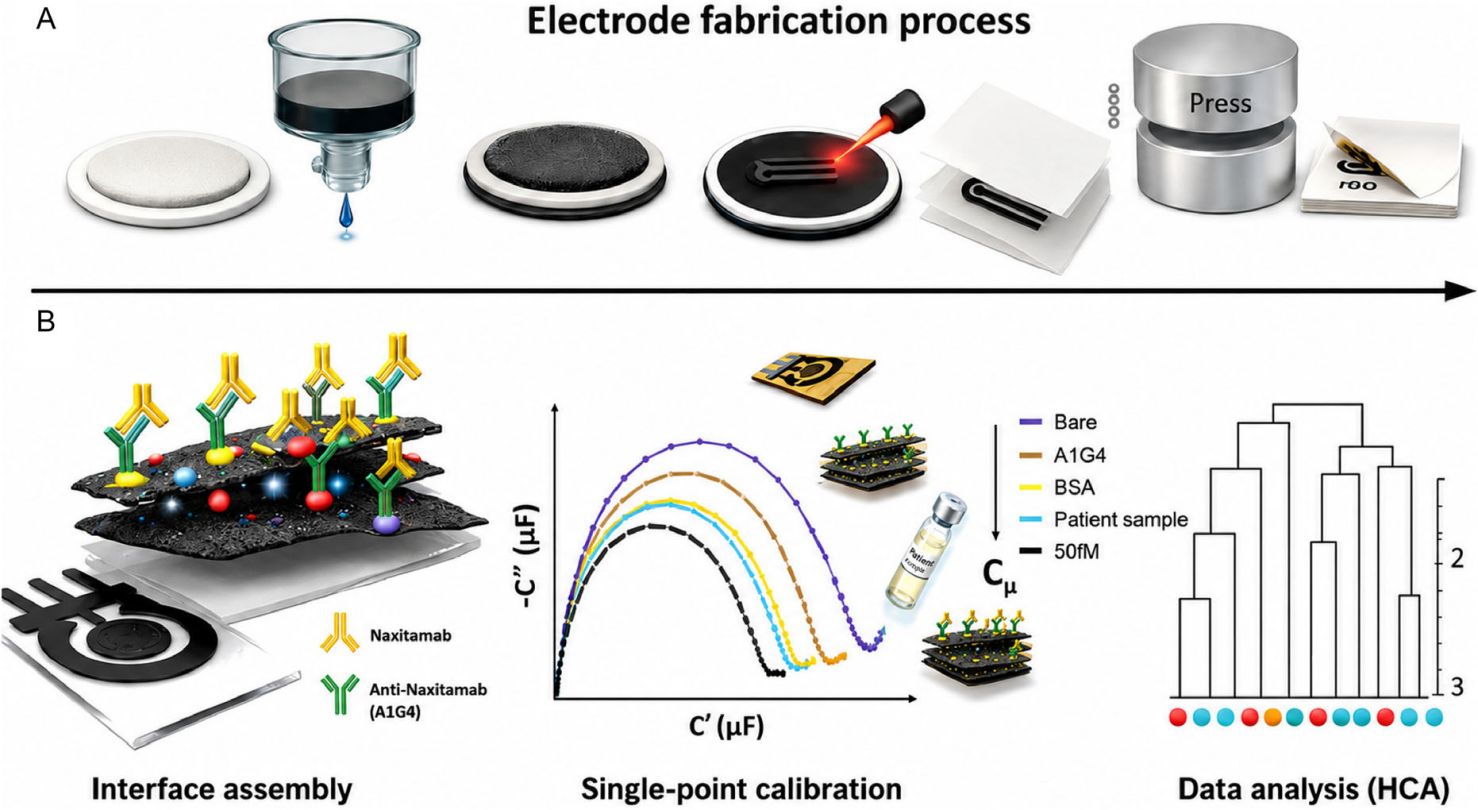
Methodological workflow from electrode preparation to biosensing and data analysis. (A) Graphical representation of the fabrication of rGO@AuNPs electrodes, including film preparation, laser engraving of the electrode pattern, and subsequent electrode stamping. (B) Schematic illustration of the biosensor assembly, including surface functionalization, blocking steps, and the specific interaction with naxitamab present in patient‐derived serum samples. The evolution of the quantum capacitance response is also depicted, showing its progressive decrease during the stepwise preparation of the biosensor interface, followed by a further concentration‐dependent decrease upon target recognition under a single‐point calibration strategy for naxitamab detection in patient samples. The resulting data are subsequently processed through hierarchical cluster analysis (HCA).

The underlying mechanism is hypothesized to involve a photothermal co‐reduction process, wherein high laser‐induced temperatures induce excitation‐state reactions that restore the *sp* ^2^ carbon network and eliminate oxygenated functional groups from the GO lattice. Simultaneously, gold cations are reduced to metallic NPs. Additionally, gas evolution and thermal expansion during the process promote film exfoliation, thereby aiding in precise patterning. The schematic representation of the fabrication process is shown in Figure 1A. The study workflow, illustrated in Figure 1B, includes biosensor assembly, quantitative analysis of real samples using a single‐point calibration strategy, and subsequent data processing for retrospective analysis of patient samples.

Although the feasibility and electrochemical performance of this fabrication approach had already been demonstrated, the present study was primarily focused on its systematic optimization. Such refinement was considered essential because the electrodes were intended for quantum capacitance measurements and for the quantification of naxitamab in real samples, applications that require highly reproducible and electrically stable platforms to ensure accurate and sensitive signal readout under clinically relevant conditions.

**FIGURE 2:**
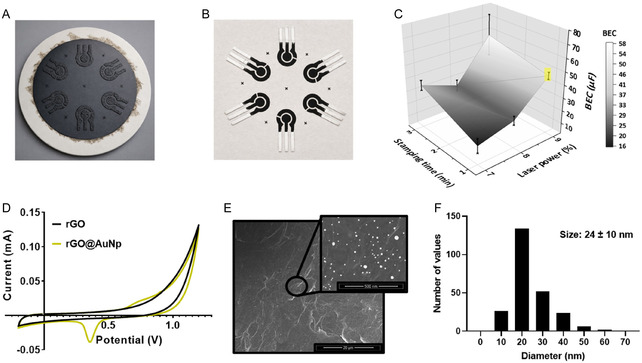
Preparation and characterization of the rGO@AuNPs electrodes. (A) GO film after laser processing, showing the patterned rGO‐based electrodes fabricated at 9% laser power. (B) Six‐electrode configuration after transfer and stamping onto a PET substrate with pre‐printed silver conductive tracks. (C) Three‐dimensional response surface representing the optimization of the fabrication parameters for rGO electrodes embedded with AuNPs. Each experimental condition was evaluated using six independently fabricated electrodes (*n* = 6). Error bars represent the mean ± standard deviation (SD). (D) Cyclic voltammetry recorded in 0.1 M sulfuric acid for rGO (black) and rGO electrodes embedded with AuNPs (yellow). (E) SEM micrograph of the rGO@AuNPs electrode surface and (F) corresponding AuNPs size distribution.

Accordingly, optimization was conducted using the bare electrode capacitance (BEC) and intra‐batch standard deviation as key performance indicators. The experiment was handled following a two‐factor factorial design, with laser power and stamping time serving as independent variables. As illustrated in Figure 2C, fabrication parameters associated with the laser patterning of rGO electrodes on GO films (Figure 2A), and their subsequent transfer and stamping onto PET substrates with pre‐printed silver tracks (Figure 2B), were systematically varied. Based on prior findings, laser powers of 7%, 8%, and 9% were selected for evaluation [9, 11]. Simultaneously, the influence of stamping conditions during electrode transfer was investigated. Consistent with Giacomelli et al.'s 2020 work, a constant pressure of 12 tons was applied, and stamping durations of 1 and 3 min were compared [12].

Analysis of the resulting BEC values and intra‐batch variability revealed that a laser power of 9% combined with a stamping time of 1 min under 12 tons of pressure provided the most favorable balance between electrical performance and reproducibility. Under these optimized conditions, electrochemical characterization exhibited profiles consistent with previous reports, with the gold reduction peak observed at approximately 0.35 V versus the rGO reference electrode (Figure 2D). The nanoparticle size distribution remained within the expected range, displaying an average diameter of 24 ± 10 nm (Figure 2E,F) [10, 11, 20]. Electrodes fabricated under these optimized conditions were subsequently employed in all further experimental procedures. The quantities of GO and gold precursor were maintained in accordance with previously validated protocols [9, 12, 19].

### 2.2 Biosensor Interface Assembly and Analytical Performance

**FIGURE 3:**
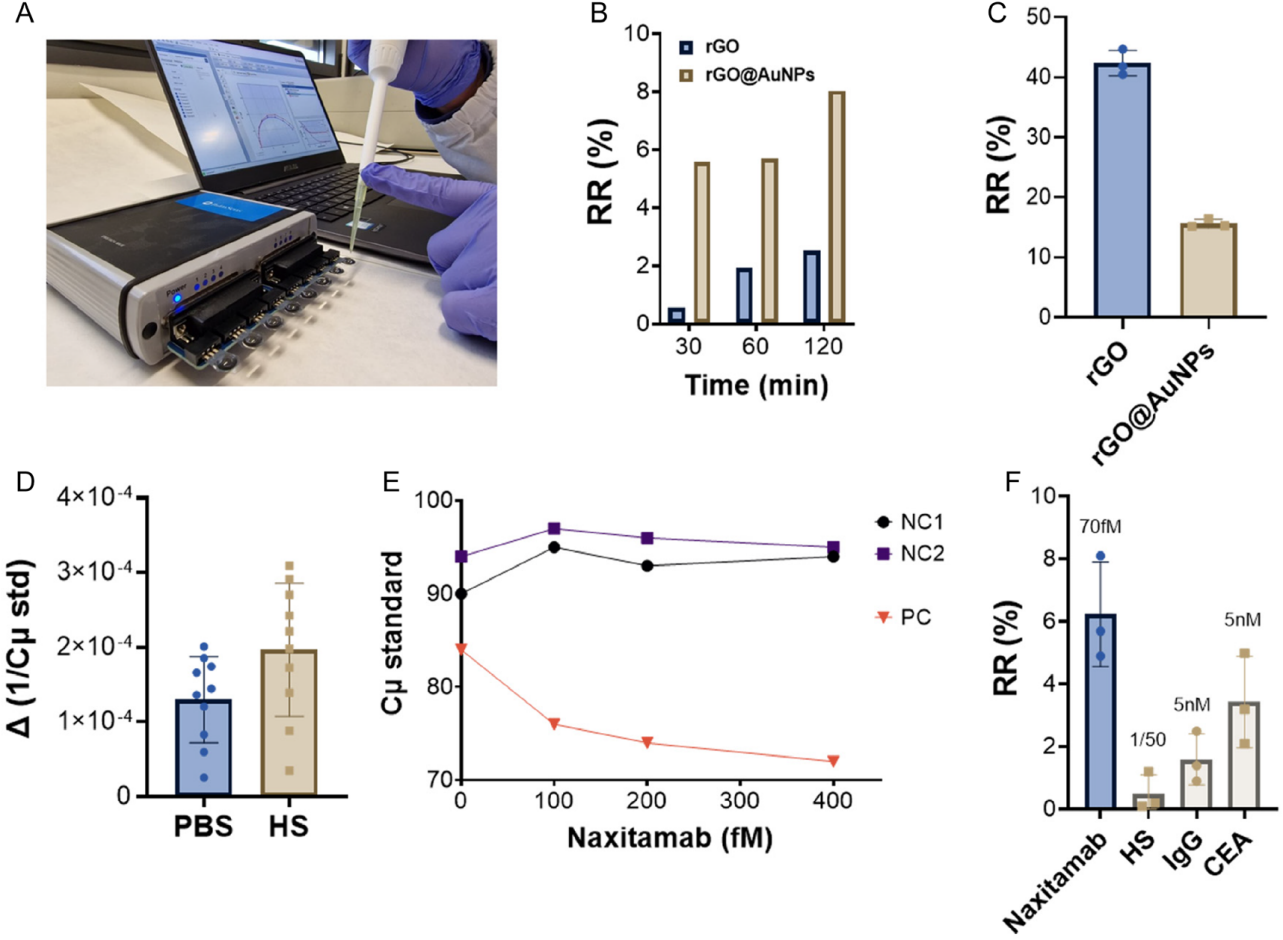
Biosensor interface assembly (A) Representation of the experimental set‐up employed for label‐free quantum capacitance measurements. (B) Kinetic study of the antibody immobilization on rGO and rGO@AuNPs electrodes using the A1G4 antibody. (C) Evaluation of the blocking efficiency after surface passivation using BSA (*n* = 3). (D) Blank response recorded in 10 independent replicates using 10 mM PBS (pH 7.4) and diluted human serum (HS, 1:1000). (E) Signal response as a function of naxitamab concentration for functionalized and nonfunctionalized electrodes (*n* = 2). (F) Specificity assessment performed using diluted human serum, IgG, and an unrelated protein (chicken egg albumin, CEA) to challenge the biosensor (*n* = 3). Error bars represent the mean ± standard deviation (SD).

After electrode fabrication and initial characterization, the assembly of the biosensor interface and its analytical performance were systematically evaluated using the experimental set‐up depicted in Figure 3A. The monoclonal antibody naxitamab A1G4 was employed as the bioreceptor in the biosensor design. Accordingly, the interaction dynamics between the antibody and the rGO@AuNPs surface were characterized to identify the optimal incubation time for immobilization. Functionalization efficiency was assessed by monitoring the change in capacitance relative to bare electrodes after 30, 60, and 120 min of antibody incubation. rGO electrodes were again used as controls to isolate the contribution of AuNPs. As shown in Figure 3B, a grouped bar chart comparing the relative response (RR, %) for rGO and rGO@AuNPs electrodes at each incubation time clearly demonstrates the enhanced response provided by the nanocomposite interface. rGO@AuNPs electrodes demonstrated a four‐ to fivefold higher functionalization efficiency compared to rGO electrodes. Moreover, functionalization kinetics were significantly accelerated in the presence of gold, likely due to thiol–gold binding interactions. In contrast, the response observed for rGO electrodes is indicative of a distinct, likely physisorptive mechanism. These results suggest a hybrid chemisorption‐physisorption mechanism may govern antibody immobilization on rGO@AuNPs surfaces.

To further substantiate the hypothesis that chemisorption is promoted by the presence of AuNPs, the blocking efficiency of bovine serum albumin (BSA) was comparatively evaluated on rGO@AuNPs and rGO electrodes, as seen in Figure 3C. The bar plot reveals a significantly higher relative response for rGO electrodes upon BSA incubation, indicating more pronounced physisorption on the rGO‐only surface. In contrast, the lower response observed for rGO@AuNPs indicates reduced physisorption compared to rGO electrodes.

It is well known that BSA contains 35 cysteine residues, of which 34 participate in 17 disulfide bridges, leaving one free thiol group available for covalent interaction with gold surfaces [21]. Nevertheless, hydrophobic interactions were found to dominate in the case of rGO‐only electrodes, which possess a higher surface area and exhibit stronger noncovalent adsorption than their rGO@AuNPs counterparts. These results are consistent with prior studies indicating relatively weak BSA binding to gold substrates [22, 23].

Despite this behavior, the interaction between BSA and rGO@AuNPs electrodes was sufficient to effectively block nonspecific adsorption sites after antibody functionalization, as demonstrated in Figure 3E. In this concentration‐dependent assay, three BSA‐blocked electrodes were evaluated in parallel: one functionalized with A1G4 as a positive control (PC) and two nonfunctionalized negative controls (NC1 and NC2). Upon exposure to increasing naxitamab concentrations, only the functionalized electrode exhibited a concentration‐dependent signal decrease, whereas both negative controls showed negligible variation. These results confirm the effectiveness of BSA as a blocking agent and validate the specificity of the antibody–antigen recognition process.

To evaluate the intrinsic background signal and matrix‐dependent variability of the platform, blank samples were prepared in phosphate‐buffered saline (PBS) and in diluted commercial human serum (1:1000 in PBS), corresponding to the matrices employed in all subsequent experimental stages (see Figure 3D). The scatter distribution and mean bars for the relative response, displayed in Figure 3D, indicate minimal variability for both matrices, with slightly higher dispersion in human serum. The normalized capacitance signals obtained from blank measurements exhibited limited dispersion in both matrices, confirming stable baseline conditions. Signal‐based limits of detection (LoD) and quantification (LoQ) were determined from blank replicates as three and ten times the standard error of the mean normalized capacitance signal, respectively. On this basis, the PBS matrix yielded signal thresholds of 5.46 × 10^−5^ for the LoD and 1.82 × 10^−4^ for the LoQ. In the human serum matrix, slightly higher signal thresholds were obtained, with LoD and LoQ values of 8.46 × 10^−5^ and 2.82 × 10^−4^, respectively, consistent with the increased baseline variability characteristic of biological media. These results were anticipated due to the presence of numerous biomolecules in human serum capable of engaging in nonspecific interactions with the biosensor, potentially modulating its electronic environment and, consequently, its quantum capacitance. These observations are in agreement with previously reported findings [11, 13, 24]. The LOD and LOQ values are reported in terms of signal variation, as the analytical response of the system is governed by electrode‐specific sensitivity under real sample conditions. In this context, signal‐based thresholds ensure that all measurements are performed within a reliable quantification regime, maintaining analytical rigor.

The biosensor's specificity was subsequently evaluated in the presence of potential interferents, including 5 nM human IgG, chicken egg albumin (CEA), and human serum diluted 1:50, twenty times more concentrated than the matrix used in calibration (see Figure 3F). The sensor response to 70 fM naxitamab exhibited an average signal change of approximately 6%, significantly exceeding those elicited by the tested interferents. Importantly, both IgG and human serum represent relevant clinical interferents, the former being universally present in patient samples and the latter serving as the actual sample matrix. Notably, the use of human serum at a 1:50 dilution represents a more demanding condition than the analytical working conditions employed for real samples (1:1000 dilution), as it involves significantly higher concentrations of endogenous proteins, including IgG. The preservation of sensor specificity under these conditions supports its robustness in clinically relevant environments. Among the individual interferents, CEA produced the highest signal shift, although still significantly lower than that of naxitamab. This protein is not endogenously found in human serum and thus does not compromise the sensor's specificity in practical applications. The elevated response to CEA is hypothesized to arise from its relatively small molecular weight (42.7 kDa), which is ~36% smaller than BSA (66.4 kDa). It is posited that CEA may occupy interstitial regions between adsorbed BSA molecules and alter the electronic environment, thereby producing a detectable signal. These findings affirm the sensor's high specificity and potential clinical applicability.

**FIGURE 4:**
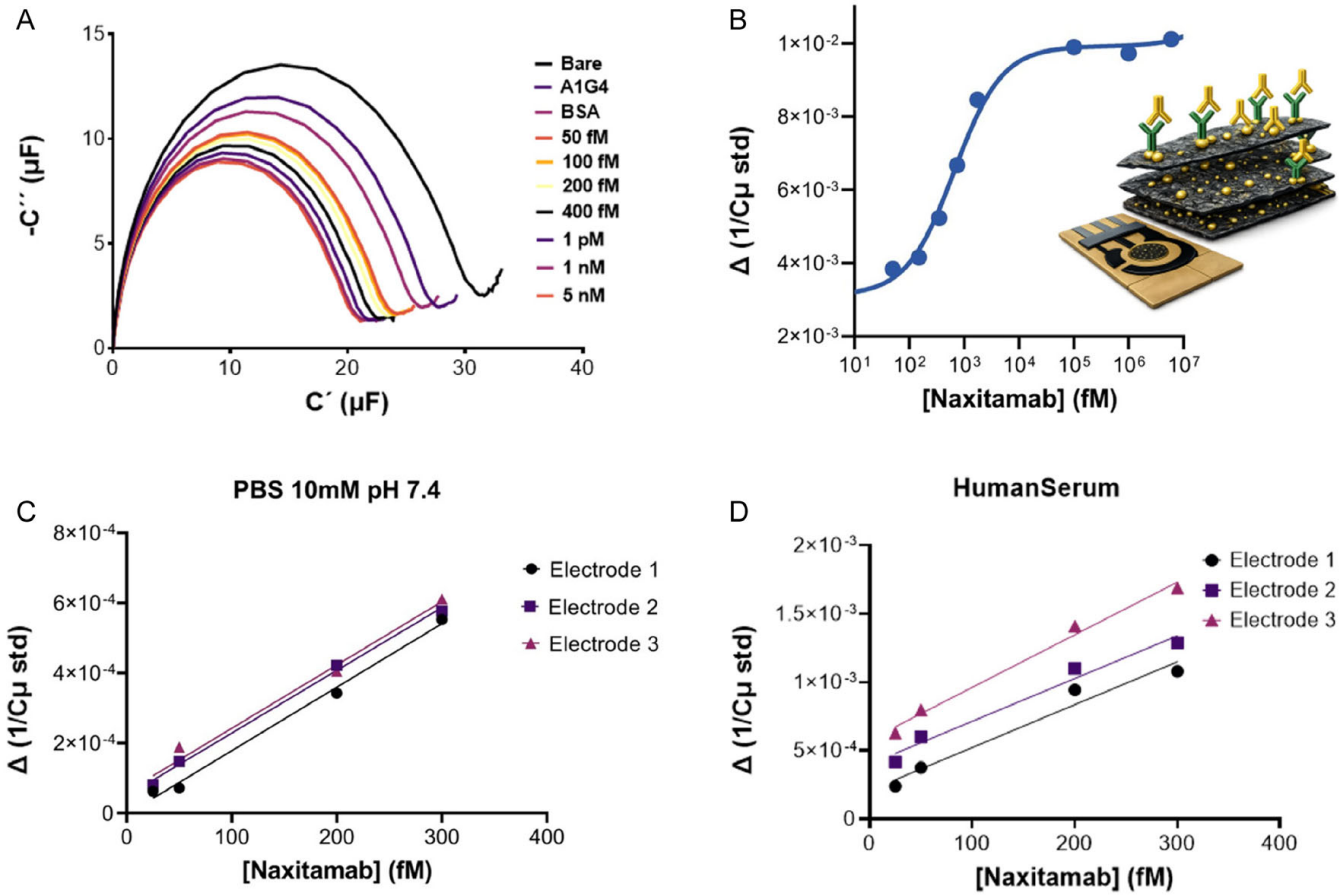
Biosensor analytical performance (A) Step‐by‐step monitoring of the biosensor fabrication and operation by capacitance measurements at each functionalization and recognition stage. (B) Binding curve for naxitamab detection obtained from the variation in capacitance, with an inset schematic illustrating the architecture of the rGO@AuNPs‐based biosensor and the antibody–antigen recognition event. (C,D) Calibration curves recorded in 10 mM PBS (pH 7.4) and human serum, respectively, using independent electrodes (*n* = 3).

Upon successful validation of biosensor specificity and functionalization, its analytical performance was investigated using calibration curves in both PBS (pH 7.4) and diluted human serum (1:1000). As illustrated in Figure 4A, stepwise additions of naxitamab to the biosensor induced a progressive reduction in the semicircular region of the Nyquist plots in the complex capacitance plane—indicative of receptor–analyte interactions. This response is consistent with established literature on quantum capacitance‐based biosensors [11, 13, 25]. A saturation trend characteristic of adsorption–limited interactions was observed in the processed calibration curve (Figure 4B), corroborating the theoretical framework underpinning this class of biosensors [11, 13, 24].

Target concentrations ranged from 50 fM to 5 nM, with a linear dynamic range identified between 50 and 300 fM, sufficient for the quantification of naxitamab in relevant clinical samples. Given the consistent response observed at 50 fM, further investigations extended the lower boundary to 25 fM. Calibration curves for PBS and human serum are shown in Figure 4C,D, respectively. For each matrix, three biosensors were evaluated within the linear range. In PBS, the biosensor response was highly reproducible across replicates. In contrast, due to inter‐electrode variability in serum, quantification was performed using a single‐point calibration strategy within the validated linear range, enabling rapid and reliable analysis of clinical samples. This framework further justifies the use of signal‐based analytical thresholds, as each electrode operates with an individual sensitivity.

In this method, each biosensor was individually calibrated using a reference sample of known concentration (50 fM), sequentially after the unknown. The analyte concentration was then calculated via cross‐multiplication based on the relative signal changes. This approach capitalized on the high individual sensitivity of each biosensor and yielded consistent results suitable for clinical applications. It is further justified to adopt a single‐point calibration approach given that the biosensor's response was rigorously demonstrated to be linear over the relevant concentration window. During method validation, calibration curves constructed in both PBS and diluted human serum exhibited a strong linearity within the 25–300 fM range, confirming that signal change is directly proportional to naxitamab concentration. Under such conditions of established linearity, a solitary reference point, selected near the anticipated analyte level, permits accurate interpolation of unknown samples by virtue of simple cross‐multiplication. This strategy inherently corrects for device‐to‐device sensitivity variation and obviates the need for full multipoint calibrations, provided that (i) the reference concentration resides well within the verified linear regime and (ii) minimal sensor drift occurs between calibration and sample measurement. Consequently, single‐point calibration delivers rapid, precise, and reproducible quantification of naxitamab, without compromising analytical rigor. Such simplification represents a key advantage for the future implementation of quantum‐capacitive biosensors in decentralized or point‐of‐care diagnostic settings.

Validation data for this quantification strategy are summarized in Table 1.

**TABLE 1:** Validation of the single‐point calibration methodology (*n* = 3).

Concentration values, fM					
*C*_t_	*C*_exp_ 1	*C*_exp_ 2	*C*_exp_ 3	Mean	SD
50	58.35	44.43	40.58	48	8
100	93.77	93.14	104.06	97	5
160	173.36	166.31	159.23	166	6

It should be noted that the present validation was performed using spiked commercial human serum. Future prospective studies should investigate whether patient‐specific matrix compositions influence the reference sensitivity employed in the single‐point calibration strategy.

Additional calibration plots, including averaged responses and corresponding error bars, are provided in the Supporting Information (Figure S1), offering a complementary statistical representation of the data.

### 2.3 Retrospective Analysis of Patient Samples

Following the validation of the single‐point calibration methodology, naxitamab concentrations were quantitatively assessed in serum samples from patients undergoing naxitamab therapy. In collaboration with the clinical team at Hospital Sant Joan de Déu in Barcelona, two sample sets were selected for analysis, comprising a total of 73 serum samples collected from 14 patients. The first covered samples from a single treatment cycle, which includes three infusions over 5 days. Blood samples were collected 5 min before and after each infusion, yielding six samples per cycle. This set was intended to characterize the short‐term pharmacokinetics of naxitamab within a treatment cycle [2]. The second set consisted of post‐infusion samples obtained at the end of each treatment cycle, extending up to cycle five in some cases. This design enabled the evaluation of long‐term pharmacokinetics across multiple cycles. To investigate intrinsic similarities among patients based on post‐infusion naxitamab levels, hierarchical clustering analysis (HCA) was implemented to objectively delineate response‐associated subgroups [26]. A complete list of patients and corresponding samples is provided in Table S1.

**FIGURE 5:**
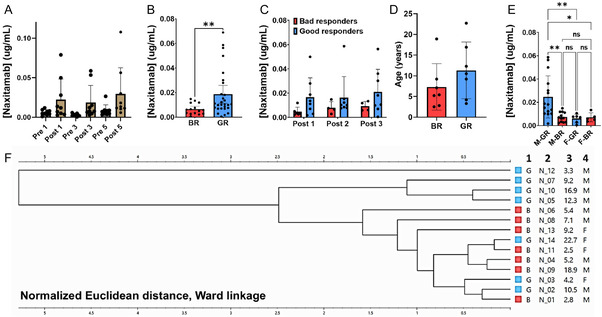
Retrospective analysis of patient samples. (A) Trend of naxitamab within cycles for patients with pre and post‐infusion samples. Clinical dataset comprising 73 serum samples from 14 pediatric neuroblastoma patients (*n* = 73 samples; *n* = 14 patients). (B) Good and bad responder (GR and BR) scores for patients with only post‐infusion samples (*n* = 43). A Welch's *t*‐test was employed to ascertain the statistical differences, yielding a *p*‐value of 0,0027. (C) Comparison of BR and GR according to naxitamab scores in post‐1, post‐3, and post‐5 inoculation samples (*n* = 38). (D) Age distribution of patients in the BR and GR groups, showing a higher mean age in the GR cohort compared to BR (*n* = 14). (E) Exploratory gender‐stratified analysis of circulating antibody levels according to therapeutic response. Antibody concentrations were compared between male good responders (M‐GR, *n* = 15), male bad responders (M‐BR, *n* = 12), female good responders (F‐GR, *n* = 6), and female bad responders (F‐BR, *n* = 5). Statistical analysis using the Kruskal–Wallis's test revealed significant differences between M‐GR and M‐BR (*P *= 0.0015), M‐GR and F‐GR (*P *= 0.0036), and M‐GR and F‐BR (*P *= 0.0138). These observations should be considered preliminary and hypothesis‐generating due to the limited cohort size. (F) Hierarchical cluster classification of patients with only post‐samples. Normalized Euclidean distance and Ward linkage were used for the classification. From left to right, the labels correspond to the following information: the patient's progression under therapy (“good evolution” G and “bad evolution” B), the patient's code, age, and gender (“male” M or “female” F). For all panels, error bars represent the mean ± standard deviation (SD). Statistical significance is denoted as follows: ns, not significant; *, one level of statistical significance; **, two levels of statistical significance.

An increase in naxitamab concentration after infusion was observed in patients for whom both pre‐ and post‐inoculation samples were available. This trend became more evident across successive treatment cycles (Figure 5A), highlighting a pharmacokinetic profile consistent with differential clinical response. When post‐inoculation samples collected at equivalent treatment stages were compared, consistently higher post‐infusion naxitamab concentrations were observed in patients classified as good responders compared with bad responders (Figure 5B). Beyond differences in mean concentrations, good and bad responder groups exhibited distinct distribution profiles. Bad responders showed a narrower concentration range clustered at lower values, whereas good responders displayed both higher median levels and increased interindividual variability, consistent with heterogeneous yet sustained systemic exposure [27].

Importantly, a substantial proportion of good responders exhibited significantly higher naxitamab concentrations than bad responders across cycles 1, 2, and 3, as shown in Figure 5C,F, a distinction which contributed to the separation observed in HCA. Across successive treatment cycles, good responders consistently maintained elevated post‐infusion naxitamab levels, whereas bad responders exhibited persistently low or nonincreasing concentrations. This divergence suggests that longitudinal concentration trends, rather than isolated measurements, may provide additional insight into therapeutic response. A noteworthy observation is that the average age of bad responders is younger than that of the group of good responders, which could be related to the previously reported higher therapeutic antibody clearance in younger children, as can be seen in Figure 5D [27, 28].

Beyond age‐related effects, a preliminary stratification of antibody levels according to therapeutic response and gender was performed (Figure 5E). A tendency toward separation between male good responders (M‐GR) and male bad responders (M‐BR) was observed, with higher circulating antibody concentrations detected in M‐GR. In contrast, no evident separation was detected between female good responders (F‐GR) and female bad responders (F‐BR), although interpretation of these trends is limited by the small number of female samples included in the present cohort. These findings should therefore be considered preliminary and hypothesis‐generating observations. Nevertheless, the observed trends may warrant further investigation in larger patient cohorts to determine whether biological sex influences systemic exposure to naxitamab or treatment response.

Finally, to investigate patterns in patient response and the factors influencing these, a HCA was performed using only post‐infusion samples. The analysis employed the normalized Euclidean distance metric with Ward linkage, a variance‐minimizing approach well suited for quantitative continuous variables and small clinical datasets [29, 30]. This combination promotes the formation of compact, well‐separated clusters, as illustrated in Figure 5F. Hierarchical clustering performed exclusively on post‐infusion naxitamab concentrations revealed patient groupings consistent with clinical evolution. Importantly, this separation was observable within early treatment cycles, underscoring the sensitivity of serum naxitamab levels to capture clinically relevant response patterns.

Although the limited number of participants precludes broad generalizations, the clustering approach allowed the detection of early response patterns within the initial treatment cycles, based on naxitamab concentrations measured 5 min post‐infusion. While certain patients deviated from the dominant clustering profiles, it is hypothesized that such discrepancies could be minimized with larger sample sizes that better reflect interpatient variability. Notably, heterogeneity of this kind is common in clinical analyses due to the inherent uniqueness of each biological sample. However, hierarchical cluster analysis (HCA) showed preliminary trends consistent with the gender‐based discrimination observed in the univariate analysis, suggesting potential differences in the clustering behavior of male and female samples.

A particularly illustrative case is patient N_06, who tested positive for HAHAs during the first treatment cycle. This patient was clearly separated from the main cluster based solely on early post‐infusion measurements, supporting the relevance of serum naxitamab levels for monitoring therapeutic response and immunogenicity. Although reduced circulating naxitamab levels may reflect immunogenicity‐associated clearance following HAHA development, the possibility that HAHAs could also influence the biosensor response cannot currently be excluded and warrants dedicated investigation in future studies.

To further visualize the clustering structure and similarity relationships among patients, multidimensional scaling (MDS) and a phylogenetic tree are presented in Section 4 of the Supporting Information (Figure S2). These complementary representations provide an alternative view of the clusters and response patterns identified by the HCA.

Collectively, these results demonstrate that rapid post‐infusion quantification of naxitamab enables early stratification of patient response profiles, captures longitudinal exposure dynamics, and provides a potential route for the indirect detection of immunogenicity, based on deviations from expected concentration patterns.

## 3 Conclusions

In this work, a label‐free electrochemical biosensor based on reduced graphene oxide electrodes embedded with AuNPs and employing quantum capacitance detection was successfully developed and optimized. The sensing platform is based on a laser‐assisted rGO@AuNPs nanocomposite, where the electronic coupling between rGO and AuNPs and the resulting nanostructured interface contribute to stable and sensitive signal transduction. In addition, the sensing strategy incorporates a single‐point calibration approach that enables quantitative analysis while avoiding conventional multipoint calibration procedures. This study demonstrates that quantum‐capacitance biosensing can be translated from material‐level innovation to clinically relevant therapeutic drug monitoring in pediatric oncology. Beyond analytical performance, this approach enables early response stratification and supports exposure‐driven therapeutic strategies.

The high specificity observed reflects the combined effect of antibody orientation on rGO‐gold nanostructures and the intrinsic sensitivity of quantum capacitance to local electronic perturbations, enabling reliable detection in complex biological matrices. Unlike conventional impedance‐based approaches, quantum‐capacitance transduction directly couples biomolecular recognition events to changes in the electronic DOS of the sensing interface, which may account for the rapid response observed in early post‐infusion measurements.

Application of the biosensor to clinical specimens provided a proof‐of‐concept demonstration of its ability to capture clinically relevant pharmacokinetic differences across treatment cycles. Hierarchical clustering of post‐infusion concentrations revealed early discrimination between good and bad responders, including the identification of patients who developed antidrug antibodies. These findings indicate that the sensitivity of the quantum‐capacitance readout is sufficient to resolve pharmacokinetic differences at early timepoints. Moreover, differences in antibody levels were observed between age groups, suggesting potential age‐related modulation of therapeutic efficacy. Preliminary trends in antibody levels were also observed according to biological sex, although these observations were limited by the modest cohort size and should be regarded as hypothesis‐generating. The observed separation of patient response profiles based solely on early post‐infusion naxitamab concentrations suggests that therapeutic exposure, rather than administered dose alone, may play a central role in treatment outcome.

This study is limited by the retrospective nature of the clinical analysis and the modest cohort size (*n* = 14 patients), although a total of 73 longitudinal serum samples were analyzed, partially mitigating cohort size limitations by enabling intra‐patient temporal assessment. While the observed trends are reliable, prospective validation in larger patient populations will be required to establish predictive clinical utility. These findings establish a proof‐of‐concept for real‐time therapeutic monitoring, which will require validation in larger prospective clinical studies.

Overall, the platform offers a rapid, minimally invasive, and point‐of‐care solution for monitoring naxitamab therapy in neuroblastoma patients. From a materials engineering perspective, the combination of laser‐assisted fabrication and rGO@AuNPs nanocomposite design provides a reproducible and scalable approach for high‐performance electrochemical interfaces. By enabling early identification of atypical exposure profiles and supporting personalized treatment strategies, this biosensor may contribute to the development of exposure‐guided therapeutic approaches. Unlike conventional ELISA‐based assays, which require centralized laboratories and extended turnaround times, the proposed platform enables near‐real‐time measurement using minimal sample volumes [31, 32, 33, 34]. With precise pipetting, sample volumes as low as 1 µL of patient serum can be employed, although the use of at least 5 µL is recommended to ensure analytical reliability and minimize volumetric errors. The single‐use design of the biosensor further supports safe clinical implementation by reducing risks of cross‐contamination and mitigating the effects of cumulative biofouling. The robust physicochemical properties of the rGO@AuNPs interface ensure stable performance during measurement, while systematic evaluation of the shelf‐life and optimal storage conditions of the fully assembled biosensor remains an important direction for future work. Prospective clinical validation and integration into hospital workflows could establish quantum‐capacitance biosensing as a new paradigm for therapeutic drug monitoring in pediatric oncology. Although the single‐point calibration strategy, combined with normalization to the BEC, effectively addresses device‐to‐device sensitivity variations, future studies would benefit from additional characterization of independently fabricated electrodes to assess the reproducibility of the electronic density‐of‐states characteristics associated with the quantum‐capacitance response. Complementary approaches, including Cµ–V profiling or area‐normalized capacitance measurements across fabrication batches, could provide useful insight for continued platform optimization and future clinical translation.

Beyond the present findings, these results provide a strong rationale for larger retrospective studies in expanded cohorts, incorporating increased patient numbers and longitudinal sampling across successive treatment cycles. Importantly, the strategy enables prospective implementation in newly treated patients, where early antibody quantification could support response stratification and real‐time dose personalization. By shifting the focus from administered dose to measured therapeutic exposure, this approach may redefine response assessment in anti‐GD2 immunotherapy. Furthermore, the modular architecture of the platform permits adaptation to other monoclonal antibodies through appropriate biorecognition elements, including aptamers, extending quantum‐capacitance biosensing to a broader spectrum of immunotherapeutic agents. Real‐time quantification of circulating antibody levels may also support refinement of pharmacokinetic models and facilitate integration into clinical workflows through rapid, decentralized feedback on therapeutic exposure.

## 4 Experimental Section

### 4.1 Materials

Graphene oxide (GO) was acquired from Global Graphene Group as a water‐based dispersion at 1 wt%. Chloroauric acid trihydrate (HAuCl_4_·3H_2_O, ≥99.9%), Immunoglobulin IgG, CEA, Human Serum, BSA, and all PBS components were purchased from Sigma–Aldrich. The monoclonal antibody A1G4, specific for naxitamab, was kindly provided by Dr. Nai‐Kong V. Cheung (Memorial Sloan Kettering Cancer Center, New York). Naxitamab was supplied by the SJD partner. Both were used without further purification. All solutions were prepared using ultrapure water (18.2 MΩ cm) obtained from a Milli‐Q system.

### 4.2 Fabrication of rGO@AuNPs Electrodes

Electrodes were fabricated following a previously validated photothermal co‐reduction strategy. Aqueous GO was mixed with HAuCl_4_ (50 mM) and deposited on PVDF membranes (pore size 0.1 µm) by vacuum filtration. The resulting films were dried at 37 °C and patterned using a CO_2_ laser (Trotec Rayjet50) with a 7.4 cm focusing lens (*λ* = 10.6 µm, power 30 W, and 0.04 mm laser spot). The maximum speed of engraving was used together with the maximum pulses per inch, 1000 PPI. Laser irradiation induced the simultaneous reduction of GO to rGO and the in situ formation of AuNPs. The patterned rGO@AuNPs films were transferred onto polyethylene terephthalate (PET) substrates pre‐printed with silver tracks by means of a hydraulic press (12 tons). Optimal performance was achieved with a laser power of 9% and a stamping duration of 1 min [35].

### 4.3 Functionalization and Blocking Protocol

Working electrode functionalization was performed by drop‐casting A1G4 antibody solution (50 µg/mL in PBS (1X), 6 µL) onto the active area, followed by incubation at 4 °C (120 min) in a humidified chamber. Unbound antibody was removed by rinsing with water. Subsequently, BSA solution (1% in PBS (1X), 5 µL) was applied for 30 min to block nonspecific adsorption sites. Electrodes were rinsed again with water before measurements. Negative control experiments were conducted using nonfunctionalized electrodes treated only with BSA.

### 4.4 Electrochemical Measurements

Capacitance measurements were performed using a Palmsens 4 potentiostat in a 3‐electrode configuration. The 3‐electrode system consisted of the rGO@AuNPs composite. All measurements were carried out in 0.1 mL of phosphate buffer (50 mM) containing KCl (100 mM) at pH 7 under ambient conditions. Electrochemical impedance spectroscopy (EIS) was performed using an AC amplitude of 0.01 V over a frequency range from 1 Hz to 10 kHz. BEC was determined at a fixed potential of 0.01 V, selected to operate the system under near‐equilibrium conditions where the response is dominated by electronic capacitance contributions and sensitivity to interfacial perturbations is maximized [11, 36]. Data were analyzed using the complex capacitance model and fitted with custom MATLAB scripts. The quantum capacitance signal was extracted from the low‐frequency range when the fitted semicircle touches the real capacitance axis. To standardize the capacitance data, the BEC was employed as the normalization factor. This method was preferred over electroactive surface area calculations based on the gold reduction peak during H_2_SO_4_ cleaning, as the latter selectively accounts for the quantum states of AuNPs, excluding their broader influence on the electrode interface. To preserve the integrity of the electrode surface and avoid potential contamination, redox probes were deliberately omitted from the characterization protocol.

### 4.5 Calibration and LoD

Calibration curves were generated by serial incubations of naxitamab standard solutions (prepared in PBS or diluted human serum). Concentration‐dependent capacitance shifts were recorded and plotted to construct calibration curves. The LoD and LoQ were calculated according to ISO 11843‐1, using the signal‐to‐noise ratio (*S*/*N* = 3 for LoD, *S*/*N* = 10 for LoQ). Single‐point calibration was implemented by referencing unknown samples to a standard solution (50 fM) that was added sequentially and analyzed under identical conditions.

### 4.6 Specificity Assays

Sensor specificity was evaluated using potential interferents, including human IgG (5 nM), chicken egg albumin (CEA, 5 nM), and human serum (1:50 dilution). Responses were compared against those elicited by naxitamab (70 fM). All samples were incubated for 30 min before measurement. Capacitance shifts were recorded and normalized to the BEC.

### 4.7 Human Serum Samples and Ethical Approval

Serum samples were collected from pediatric patients undergoing naxitamab therapy at Hospital Sant Joan de Déu (Barcelona, Spain), in accordance with the Helsinki Declaration and in compliance with the Hospital Sant Joan de Déu Research Ethics Committee (CEIm) under approval number PS‐11‐22. Informed consent was obtained from all patients’ legal guardians. Samples were collected 5 min before and after naxitamab infusion at multiple treatment cycles or at the end of each cycle. Each sample was aliquoted, anonymized, and stored at −80 °C until analysis. No freeze–thaw cycles were performed before testing.

Clinical responses were assessed following the International Neuroblastoma Response Criteria (INRC) after 2 cycles of anti‐GD2 treatment. Good Responders (GR) accounted for patients who achieved any objective response (complete remission or partial response) or disease control (stable disease), and those classified as Bad Responders (BR) corresponded to patients who progressed under treatment.

Samples were collected during standard clinical administration of the antibody in accordance with current therapeutic protocols. Naxitamab monotherapy is delivered as a 30–60 min intravenous infusion in outpatient settings, in 4 week cycles, to patients with relapsed/refractory high‐risk neuroblastoma limited to bone or bone marrow. For patients with chemoresistant or monotherapy‐refractory disease, naxitamab chemoimmunotherapy following the HITS protocol is used, combining naxitamab with irinotecan, temozolomide, and GM‐CSF. For the naxitamab chemoimmunotherapy protocol, the total dose of naxitamab per cycle is 9 mg/kg, the same as naxitamab monotherapy.

### 4.8 Retrospective Analysis and Clustering

Naxitamab concentrations were determined in pre‐ and post‐infusion serum samples using the single‐point calibration strategy. Prior to analysis, all clinical serum samples were diluted 1:1000 in PBS, matching the conditions used for calibration standards. Data were processed using MATLAB R2024b and Orange Data Mining 3.38.1, and clustered using HCA, based on the normalized Euclidean distance and Ward's linkage. To facilitate visualization of the similarity relationships among patients and to support the interpretation of the clustering structure, MDS was additionally applied to project the samples into a two‐dimensional space while preserving pairwise distances between observations.

### 4.9 Data Analysis

Electrochemical capacitance values were extracted using a custom MATLAB script that converts impedance data into the capacitive Nyquist representation (−*C*″ vs. *C*′) and parameterizes the frequency‐dependent response through circular fitting. The complex capacitance was defined as $C^{*}(\omega )=C\prime (\text{ω})-jC\prime \prime (\text{ω})$ and obtained from the impedance according to $C^{*}\left(\omega \right)=\frac{1}{j\omega Z^{*}\left(\omega \right)}$. The script uses the measured impedance magnitude (|*Z*|) to implement a numerically stable form of this conversion, minimizing error propagation at low frequencies [11, 15]. The fitted semicircle radius was used as a quantitative descriptor of the electrochemical capacitance. In addition to calibration‐based analysis, the same script enabled direct estimation of analyte concentration in real samples from relative changes in the fitted capacitance response, as detailed in Supporting Information (Section 3).

## Author Contributions

**Andy Bruno**: writing – original draft, review and editing, investigation, formal analysis, data curation, methodology, conceptualization. **R** **uslán** **Alvarez‐Diduk**: writing – review and editing, supervision, resources, project administration, methodology, conceptualization. **Paula Lara**: investigation, formal analysis. **Gabriel Maroli**: writing, data curation, MATLAB programming. **Cristina Larrosa**: selection of patients, clinical advice, data curation. **Sandra López‐Miralles**: sample collection. **Jaume Mora**: conceptualization, sample collection, funding acquisition. **Carlos J. Rodríguez‐Hernández**: writing – review and editing, supervision, resources, project administration, methodology, conceptualization, funding acquisition. **A** **rben** **Merkoçi**: writing – review and editing, validation, supervision, funding acquisition.

## Funding

This study was supported by the CERCA programme/Generalitat de Catalunya. The ICN2 is supported by the Severo Ochoa Centres of Excellence programme (Grant CEX2021‐001214‐S), funded by MCIN/AEI/10.13039.501100011033. Project AC21_2/00044, funded by Instituto de Salud Carlos III (ISCIII) and co‐funded by the European Union Next Generation EU/PRTR (GLEBioassay). The ICN2 is funded by the CERCA programme/Generalitat de Catalunya. This project has received funding from the European Union's Horizon Europe – the Framework Programme for Research and Innovation (2021–2027) under grant agreement No 101120706.

## Conflicts of Interest

The authors declare no conflicts of interest.

## Supporting information

Supplementary Material

## Data Availability

The data will be available in the CORA – Catalan Open Research Area at the following DOI: https://doi.org/10.34810/DATA3517.
