# Supplementary material for: Quantum‐Capacitance Biosensing Enables Real‐Time Monitoring of Naxitamab for Therapeutic Response Stratification in Neuroblastoma
Source: Small Sci. 2026 Jul 31;6(8):e70349. doi: 10.1002/smsc.70349 (PMC13431878; doi:10.1002/smsc.70349)
Supplement: Supplementary file 1 — Supplementary Material [file SMSC-6-e70349-s001.pdf]

“Quantum-capacitance biosensing enables real-time monitoring of naxitamab for therapeutic response stratification in neuroblastoma.”

Andy Bruno[1,2], Ruslán Alvarez-Diduk[2*], Paula Lara[2], Gabriel Maroli[2], Cristina Larrosa[3], Sandra López-Miralles[3], Jaume Mora[3], Carlos J. Rodríguez-Hernández[4], Arben Merkoçi[2,5*]

[1]Universitat Autonoma de Barcelona (UAB), Bellaterra 08193, Barcelona, Spain.

[2]Catalan Institute of Nanoscience and Nanotechnology (ICN2), CSIC and BIST, Campus UAB, Bellaterra, 08193 Barcelona, Spain.

[3]Pediatric Cancer Center Barcelona (PCCB), Hospital Sant Joan de Déu, 08950 Esplugues de Llobregat, Spain.

[4]Pediatric Cancer, Institut de Recerca Sant Joan de Déu (IRSJD), 08950 Esplugues de Llobregat, Spain.

[5]Catalan Institution for Research and Advanced Studies (ICREA) Passeig de Lluís Companys, 23, Barcelona, 08010, Spain.

*** Correspondence:** ruslan.alvarez@icn2.cat, arben.merkoci@icn2.cat

Keywords: laser-assisted | rGO-based composite | label-free quantum-capacitance biosensor | single-point calibration | therapeutic drug monitoring | neuroblastoma | Naxitamab

Section 1: Mean Naxitamab Concentrations and Pre-Assigned Clinical Labels

Table S1 summarizes the mean naxitamab concentration and corresponding standard deviation, calculated from three independent measurements for each clinical sample included in the study. In addition to the quantitative values, the table lists the clinical metadata assigned to each patient prior to hierarchical clustering analysis. These metadata include the clinical response category and other relevant descriptors used to contextualize the clustering results.

Together, these data provide the quantitative and contextual framework for the classification shown in Figure 5, enabling direct comparison between measured naxitamab levels and predefined clinical characteristics, as well as facilitating the interpretation of clustering patterns and the distinction between responder groups discussed in the main text.

Table S1: Mean Naxitamab Concentrations (n = 3) and Pre-Assigned Clinical Labels

Patient	Sample code	Time	[Naxitamab] µg/mL	SD µg/mL	Age at sample	Evolution	Gender
N_04	BASAL	0	0.00085	0.00007	5.2	B	M
N_04	C1	POST	0.0112	0.0009	5.2	B	M
N_04	C2	POST	0.009	0.001	5.2	B	M
N_04	C3	POST	0.0069	0.0003	5.2	B	M
N_04	C4	POST	0.01	0.0004	5.2	B	M
N_11	C2D1	PRE	0.0048	0.0003	2.5	B	F
N_11	C2D1	POST	0.005	0.0005	2.5	B	F
N_11	C2D3	PRE	0.0029	0.0003	2.5	B	F
N_11	C2D3	POST	0.0076	0.0003	2.5	B	F
N_11	C2D5	PRE	0.0089	0.0003	2.5	B	F
N_11	C2D5	POST	0.013	0.001	2.5	B	F
N_06	C1	POST	0.0022	0.0009	5.4	B	M
N_10	C2D1	PRE	0.008	0.0003	16.9	G	M
N_10	C2D3	PRE	0.00048	0.00005	16.9	G	M
N_10	C2D5	PRE	0.0091	0.0005	16.9	G	M
N_10	C2D1	POST	0.014	0.002	16.9	G	M
N_10	C2D3	POST	0.012	0.002	16.9	G	M
N_10	C2D5	POST	0.03	0.001	16.9	G	M
N_12	C2D1	PRE	0.012	0.004	3.3	G	M
N_12	C2D1	POST	0.05	0.002	3.3	G	M
N_12	C2D3	PRE	0.004	0.001	3.3	G	M
N_12	C2D3	POST	0.059	0.006	3.3	G	M
N_12	C2D5	PRE	0.027	0.002	3.3	G	M
N_12	C2D5	POST	0.056	0.003	3.3	G	M
N_07	C2D1	PRE	0.0072	0.0002	9.2	G	M
N_07	C2D1	POST	0.028	0.002	9.2	G	M

N_07	C2D3	PRE	0.0059	0.0003	9.2	G	M
N_07	C2D3	POST	0.015	0.001	9.2	G	M
N_07	C2D5	PRE	0.007	0.0001	9.2	G	M
N_07	C2D5	POST	0.018	0.002	9.2	G	M
N_09	C2D1	PRE	0.0049	0.0003	18.9	B	M
N_09	C2D1	POST	0.0119	0.0003	18.9	B	M
N_09	C2D3	PRE	0.00195	0.00002	18.9	B	M
N_09	C2D3	POST	0.0065	0.0007	18.9	B	M
N_09	C2D5	PRE	0.0047	0.0005	18.9	B	M
N_09	C2D5	POST	0.0118	0.0006	18.9	B	M
N_03	basal	0	0.0014	0.0003	4.2	G	F
N_03	C1.1	post	0.0029	0.0001	4.2	G	F
N_03	C2	post	0.0079	0.0003	4.2	G	F
N_03	C3	post	0.00047	0.00006	4.2	G	F
N_03	C4	post	0.069	0.002	4.2	G	F
N_03	C5	post	0.0031	0.0004	4.2	G	F
N_01	C1	post	0.0033	0.0005	2.8	B	M
N_01	C2	post	0.0149	0.0002	2.8	B	M
N_01	C3	post	0.0062	0.0008	2.8	B	M
N_01	C4	post	0.00066	0.00002	2.8	B	M
N_05	basal	0	0.0031	0.0002	12.3	G	M
N_05	C1	post	0.019	0.003	12.3	G	M
N_05	C2	post	0.0102	0.0006	12.3	G	M
N_05	C3	post	0.04	0.02	12.3	G	M
N_05	C4	post	0.0069	0.0004	12.3	G	M
N_08	C2D1	pre	0.0009	0.0008	7.1	B	M
N_08	C2D1	post	0.0026	0.0002	7.1	B	M
N_08	C2D3	pre	0.0028	0.0009	7.1	B	M
N_08	C2D3	post	0.0029	0.0002	7.1	B	M
N_08	C2D5	pre	0.003	0.001	7.1	B	M
N_08	C2D5	post	0.11	0.03	7.1	B	M
N_13	C1D1	pre	0.00302	0.00005	9.2	B	F
N_13	C1D1	post	0.00352	0.00007	9.2	B	F
N_13	C1D3	pre	0.0037	0.0001	9.2	B	F
N_13	C1D3	post	0.05	0.02	9.2	B	F
N_13	C1D5	pre	0.0084	0.0003	9.2	B	F
N_13	C1D5	post	0.0059	0.0004	9.2	B	F
N_14	C3D1	pre	0.0043	0.0007	22.7	G	F

N_14	C3D1	post	0.007	0.003	22.7	G	F
N_14	C3D3	pre	0.005	0.0004	22.7	G	F
N_14	C3D3	post	0.0076	0.0004	22.7	G	F
N_14	C3D5	pre	0.0051	0.0002	22.7	G	F
N_14	C3D5	post	0.011	0.002	22.7	G	F
N_02	basal	0	0.0015	0.0001	10.5	G	M
N_02	C1	post	0.0018	0.0007	10.5	G	M
N_02	C2	post	0.01	0.002	10.5	G	M
N_02	C3	post	0.0099	0.0007	10.5	G	M

Section 2: Calibration Reproducibility and Statistical Validation

To complement the individual calibration curves presented in the main manuscript, additional calibration plots are provided here in the form of averaged responses with corresponding standard deviations (n = 3 independent electrodes). These representations allow for a conventional statistical evaluation of the analytical performance while maintaining consistency with the electrode-to-electrode variability discussed in the main text.

Approximate concentration-domain LoD and LoQ values were additionally estimated by inverse prediction from blank-derived signal thresholds. Briefly, the signal-domain LoD and LoQ thresholds were defined as the mean blank response plus three and ten times the standard deviation of the blank measurements, respectively, and were subsequently converted into concentration units using the corresponding calibration equations. Under human serum conditions, one of the three individual electrode-specific calibration models yielded positive concentration-equivalent LoD estimates (25 fM), whereas two models produced a mathematically negative inverse-predicted LoD. This occurred because the signal-domain detection threshold remained lower than the fitted calibration intercept. Since negative analyte concentrations have no physical meaning, these results indicate that the corresponding detection thresholds lie below the physically meaningful concentration range defined by those calibration models.

The concentration-domain LoQ values exhibited substantial variability across the three individual calibration models, yielding values of approximately 24, 86, and 148 fM, corresponding to a mean LoQ of approximately 86 fM. In contrast, the LoQ estimated from the averaged calibration curve was approximately 82 fM. This dispersion reinforces the rationale behind the single-point normalization strategy adopted in this work, where each biosensor is treated as an individually normalized sensing interface rather than as part of a globally transferable calibration population.

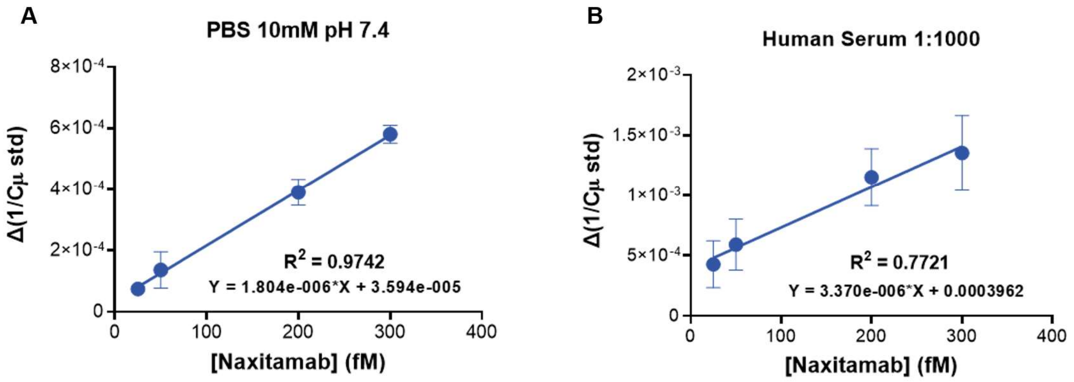

Figure S1. Calibration curves for naxitamab detection in (A) PBS and (B) human serum, presented as mean response ± standard deviation (n = 3 independent electrodes). These representations complement Figures 4C and 4D, where individual calibration curves are shown to highlight electrode-to-electrode variability. The averaged data confirm the linearity of the system within the working range, while also reflecting the variability observed between independently fabricated biosensors.

It should be noted that these LoD and LoQ values were obtained from the specific calibration models shown in Figure S1, whereas clinical samples were quantified using independently calibrated biosensors following the electrode-specific single-point calibration strategy described in the main

manuscript. Consequently, the concentrations reported in Table S1 were not derived from the same calibration models used for the present LoD and LoQ estimations.

For example, the lowest naxitamab concentration reported in the clinical cohort corresponds to patient N_03 (cycle 3), with an estimated concentration of approximately 32 fM. This concentration was first determined through the electrode-specific single-point calibration approach by applying proportional quantification relative to the 50 fM reference standard and was subsequently expressed as 0.00047 µg / mL after correction for the dilution factor and conversion using the molecular weight of naxitamab. Although this concentration lies above the LoQ estimated for one calibration model and below those estimated for the remaining models, this observation reflects the variability among independently calibrated biosensors and is consistent with the electrode-specific quantification strategy employed throughout the study.

Furthermore, all clinical samples were analyzed in triplicate using three independently fabricated biosensors, with each replicate quantified through its corresponding electrode-specific calibration procedure. For the N_03 cycle 3 sample, the three independent determinations yielded concentrations of 0.00047, 0.00054, and 0.00041 µg mL^{-1}. The close agreement among these independently calibrated measurements further supports the robustness of the reported concentration estimates despite the electrode-to-electrode variability observed under serum conditions.

Taken together, these observations provide additional context for interpreting the concentration-domain LoD and LoQ values reported above and further support the implementation of electrode-specific calibration procedures throughout the analytical workflow.

Section 3: Processing of capacitive EIS data and radius-based quantification from capacitive Nyquist plots

Electrochemical impedance spectroscopy (EIS) datasets were post-processed in MATLAB (MathWorks, USA) to quantify concentration-dependent changes in the capacitive response. In addition to conventional impedance representations, the analysis focused on the capacitive Nyquist plane, i.e., the imaginary capacitance component plotted versus the real capacitance component (-C′′ vs. C′), as commonly used in the analysis of capacitive biosensing interfaces. In this representation, the interfacial charging process typically yields a semicircular trajectory over frequency.

Raw EIS data were imported from multi-sheet Excel files. Each sheet corresponded to a distinct condition or analyte concentration. Sheets were automatically labeled by parsing the numeric prefix in the sheet name (e.g., 10 pM); special cases such as Bare and BSA were detected case-insensitively. For each sheet, rows containing missing values in the relevant columns were discarded. The complex capacitance was defined as $C^*(\omega) = C'(\omega) - jC''(\omega)$. Real and imaginary capacitance components were then computed pointwise from the impedance outputs using frequency (f), impedance magnitude (|Z|), and the real/imaginary impedance components, following the standard conversion from complex impedance to complex capacitance:

$$C^*(\omega) = \frac{1}{j\omega Z^*(\omega)}$$

with $C' = -Z''/\omega|Z|^2$ and $C'' = Z'/\omega|Z|^2$, where $\omega = 2\pi f$ and $j = \sqrt{-1}$. The script uses the measured impedance magnitude (|Z|) to implement a numerically stable form of this conversion, minimizing error propagation at low frequencies. [1,2]

To robustly estimate the characteristic semicircle size in the capacitive Nyquist plane, the point cloud (C′, -C′′) for each condition was fitted to a circle using a least-squares algebraic circle fit. Because experimental data may contain outliers (e.g., low-frequency drift, wiring or contact artifacts, or unstable points), the fitting procedure included an iterative outlier-rejection routine. After an initial fit, the coefficient of determination (R^2) was computed, as a goodness-of-fit metric, by comparing the measured -C′′ values to the corresponding -C′′ predicted by the fitted circle. If R^2 was below a predefined threshold (0.99), the point with the largest radial residual (absolute deviation from the fitted radius) was removed and the fit repeated. This process continued until $R^2 \geq 0.99$ or a maximum number of iterations was reached. The final fitted radius (R) was used as the scalar descriptor of the semicircle size for that condition.

For datasets containing a bare-electrode measurement as a reference, fitted diameters (D = 2R) were normalized to the bare condition and transformed using an inverse-diameter metric, which yields a positive calibration slope, thereby facilitating visualization and interpretation of the concentration-dependent trend. Linear regression of the transformed metric versus concentration was performed to obtain the calibration slope and intercept, together with the corresponding R^2.

For datasets without a bare-electrode measurement as a reference (real samples), the script reports concentration estimates based on the relative changes in fitted radii between reference conditions and the unknown sample, using the inverse-radius formulation.

Section 4: Supplementary Analysis of Patient Stratification and Naxitamab Distribution

Multidimensional scaling (MDS) was applied to project patient samples into a two-dimensional space while preserving pairwise distances between observations (Figure S2A). Each point represents an individual patient and is coloured according to clinical response category (bad responders, red; good responders, blue), while marker shape indicates patient gender (circles, female; crosses, male). The background colour gradient represents the estimated class regions derived from the local neighbourhood structure in the projected space. The low Kruskal stress value (0.077) indicates that the two-dimensional representation accurately preserves the distance relationships among samples.[3] A general tendency toward separation between response groups is observed, consistent with the clustering pattern identified by hierarchical cluster analysis (HCA). Within the group of good responders, a partial separation according to gender appears to emerge, with most male patients occupying a similar region of the projection, with the exception of patient N_02, which lies closer to the distribution of female patients. In addition, the projection corroborates that patient N_06, who developed HAHAs, appears separated from the remaining bad responders, in agreement with the pattern observed in the HCA analysis.

To further facilitate the interpretation of these similarity relationships, a phylogenetic tree representation derived from the hierarchical clustering analysis (Euclidean distance, Ward's linkage) is also shown in Figure S2B. This complementary visualization highlights the clustering structure and supports the response-related grouping observed in the MDS projection.

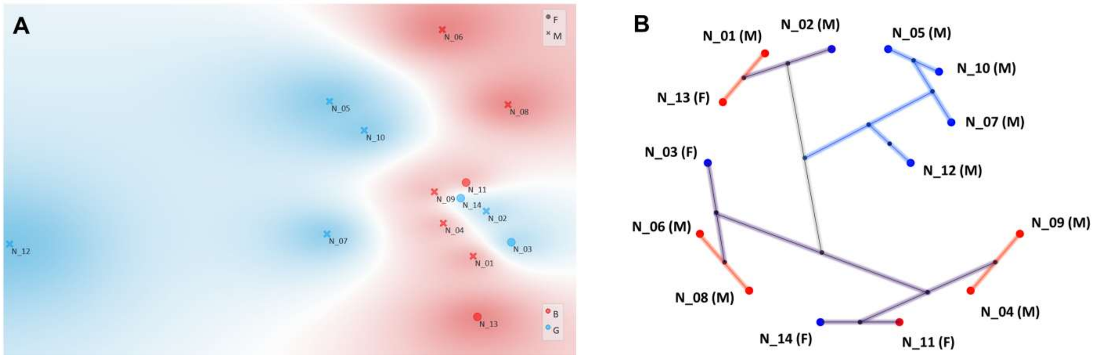

Figure S2. **Multidimensional scaling and phylogenetic tree representation of patient samples analysis. (A)** Patient samples are projected into a two-dimensional space while (Kruskal stress value: 0.077) preserving pairwise distances between observations. Points are coloured according to clinical response (bad responders, red; good responders, blue), and marker shape indicates patient gender (circles, female; crosses, male). The background colour gradient represents estimated class regions based on the local neighbourhood structure. **(B)** The phylogenetic tree provides an alternative visualization of the similarity relationships among patients derived from hierarchical clustering (Euclidean distance, Ward's linkage), highlighting the clustering structure and response patterns identified in the dataset. Patient labels indicate the patient identifier followed by gender (M, male; F, female), while marker colour denotes clinical response (red, bad responders; blue, good responders).

The fidelity of the hierarchical clustering structure was evaluated using the cophenetic correlation coefficient, which yielded a value of 0.94, indicating a strong agreement between the original

pairwise distance matrix and the dendrogram structure, suggesting that the clustering procedure preserves the similarity structure of the data with minimal distortion.[4,5]

References:

[1] B. Lucas Garrote, L. C. Lopes, E. F. Pinzón, F. C. Mendonça-Natividade, R. B. Martins, A. Santos, E. Arruda, P. R. Bueno, "Reagentless Quantum-Rate-Based Electrochemical Signal of Graphene for Detecting SARS-CoV-2 Infection Using Nasal Swab Specimens" *ACS Sens.* **2022**, *7*, 2645–2653.

[2] D. Echeverri, E. Calucho, J. Marrugo-Ramírez, R. Álvarez-Diduk, J. Orozco, A. Merkoçi, "Capacitive immunosensing at gold nanoparticle-decorated reduced graphene oxide electrodes fabricated by one-step laser nanostructuration" *Biosens. Bioelectron.* **2024**, *252*, 116142.

[3] J. B. Kruskal, "Multidimensional Scaling by Optimizing Goodness of Fit to a Nonmetric Hypothesis" *Psychometrika* **1964**, *29*, 1–27.

[4] R. R. Sokal, F. J. Rohlf, "The comparison of dendrograms by objective methods" *Taxon* **1962**, *11*, 33–40.

[5] J.-P. Brunet, P. Tamayo, T. R. Golub, J. P. Mesirov, "Metagenes and molecular pattern discovery using matrix factorization" *Proceedings of the National Academy of Sciences* **2004**, *101*, 4164–4169.
